# A novel antibacterial-immunomodulatory injectable hydrogel for periodontitis management: an in vitro study

**DOI:** 10.1186/s12903-026-09158-w

**Published:** 2026-07-07

**Authors:** Ce Bian, Xinmiao Lan, Yuqing Zhang, Miao Liu, Ruixuan Li, Bo Wang, Ning Zhang, Li Cao

**Affiliations:** 1 Department of Orthodontics, Beijing Stomatological Hospital and School of Stomatology, Capital Medical University, No. 9 Fanjiacun Road, Fengtai District, Beijing, 100070 PR China; 2 Beijing Area Major Laboratory of Peptide and Small Molecular Drugs, Beijing Laboratory of Biomedical Materials, School of Pharmaceutical Sciences, Capital Medical University, Beijing, China; 3 Department of Stomatology, The First Affiliated Hospital of Dalian Medical University, No 222 zhongshan Road, Dalian, Liaoning 116000 China

**Keywords:** Hydrogel, Apremilast, DMAHDM, Antibacterial activity, Periodontitis

## Abstract

**Background:**

Considering the limitations of current therapies for periodontitis, this study aimed to design and develop a multifunctional injectable hydrogel based on the pathogenesis of periodontitis. Its biocompatibility, antibacterial, anti-inflammatory and immunomodulatory properties were evaluated in vitro, and its potential for clinical application in periodontitis treatment was further explored.

**Methods:**

The phosphodiesterase-4 (PDE4) inhibitor Apremilast (Apr) and the quaternary ammonium antibacterial agent dimethylaminohexadecyl methacrylate (DMAHDM) were incorporated into methacrylated sodium alginate solutions at varying ratios and thoroughly mixed. A photoinitiator was then added and the mixture was exposed to 405 nm light for 20 s to induce hydrogel formation. The resulting hydrogel extracts were subsequently evaluated for their biocompatibility, antibacterial activity, anti-inflammatory effects and immunomodulatory function.

**Results:**

The hydrogel containing 50 µM Apr and 5 µg/mL DMAHDM exhibited excellent biocompatibility and effectively inhibited the growth of the periodontal pathogens *Porphyromonas gingivalis* and *Fusobacterium nucleatum in vitro*. qPCR and ELISA revealed that the hydrogel significantly downregulated the mRNA expression of the pro-inflammatory cytokines TNF-α and IL-6, as well as their corresponding protein levels. Moreover, flow cytometry analysis showed that the hydrogel displayed immunomodulatory activity by regulating macrophage polarization, indicating potential anti-inflammatory and immunoregulatory effects.

**Conclusions:**

This novel injectable multifunctional hydrogel demonstrates promising potential for clinical application, offering a new therapeutic strategy for periodontitis treatment.

## Background

Periodontitis is a common chronic inflammatory disease that affects more than one billion people worldwide [1]. Its pathogenesis is complex and primarily involves the colonization of pathogenic bacteria in dental plaque biofilms, along with dysregulation of the host immune response, leading to persistent inflammation and destruction of periodontal tissues. This process can lead to alveolar bone resorption, tooth mobility, and even tooth loss, thereby significantly impairing oral function and quality of life [2]. In recent years, periodontitis has shown a clear trend toward earlier onset, likely influenced by modern lifestyle factors such as work-related stress and circadian rhythm disturbances [3]. Therefore, developing precise intervention strategies based on its pathogenesis is of substantial clinical significance.

Currently, conventional treatments for periodontitis primarily rely on mechanical debridement, including supragingival scaling and subgingival curettage, often in combination with systemic or local antibiotics for infection control [4]. However, pathogenic bacteria within periodontal pockets often reside in anatomically complex and inaccessible areas, making complete mechanical removal of plaque difficult and leaving residual microbial populations that may contribute to disease recurrence. Local antimicrobial agents, such as chlorhexidine, can temporarily inhibit bacterial growth; however, their efficacy is limited by the dynamic oral environment, which makes it difficult to maintain stable therapeutic concentrations. Prolonged use may also disrupt microbial balance and contribute to the development of antibiotic resistance [5]. Although systemic antibiotics possess broad-spectrum antibacterial activity, their long-term use is limited by the risk of resistance and adverse systemic effects, including disruption of the gut microbiota [6].

Cyclic adenosine 3’,5’-monophosphate (cAMP) is an important intracellular second messenger synthesized from adenosine triphosphate by adenylyl cyclase and involved in cellular signal transduction as well as the regulation of diverse physiological processes [7]. In recent years, cAMP-mediated signaling pathways have attracted increasing attention for their potential role in the treatment of periodontitis, although research in this area remains limited. Previous studies have shown that oral administration of phosphodiesterase (PDE) inhibitors can alleviate alveolar bone resorption and inflammation by elevating intracellular cAMP levels, suggesting a crucial regulatory role for cAMP signaling in the pathogenesis of periodontitis [8]. However, systemic administration of PDE inhibitors often induces gastrointestinal adverse effects, which restrict their clinical application. Therefore, the development of locally targeted delivery systems for PDE inhibitors to precisely modulate cAMP signaling holds significant research value.

Currently, antibacterial therapy for periodontitis primarily relies on mechanical debridement combined with antibiotics. However, factors such as the protective barrier formed by biofilms, rapid drug clearance and the emergence of resistance make it difficult to maintain effective local drug concentrations, thereby limiting therapeutic efficacy and safety [9]. In recent years, quaternary ammonium antibacterial agents have attracted considerable attention due to their broad-spectrum and highly efficient antimicrobial properties. These agents exert their effects through electrostatic interactions between positively charged quaternary ammonium groups and negatively charged bacterial cell membranes, thereby disrupting membrane integrity and causing leakage of intracellular contents. Furthermore, their long alkyl chains can penetrate bacterial membranes, enhancing bactericidal activity and providing sustained antimicrobial effects [10]. As a novel quaternary ammonium antibacterial agent, dimethylaminohexadecyl methacrylate (DMAHDM) exhibits broad-spectrum and sustained activity against both Gram-positive and Gram-negative bacteria [11]. It has been successfully incorporated into orthodontic materials, composite resins and dentin adhesives, with its potent and stable antimicrobial properties well reported.

Injectable hydrogels, owing to their excellent plasticity, biocompatibility, and controllable gelation properties, can be minimally invasively delivered into periodontitis-affected sites and undergo in situ gelation, enabling precise filling of irregular periodontal defects. Moreover, they can serve as local drug delivery systems, enhancing therapeutic efficacy while minimizing systemic adverse effects [12]. In recent years, multifunctional hydrogels combining antibacterial, anti-inflammatory, and tissue-regenerative functions have attracted increasing attention for their ability to precisely modulate the periodontal microenvironment. Building on the anti-inflammatory effects of PDE inhibitors mediated by cAMP signaling and the potent antibacterial activity of the quaternary ammonium compound DMAHDM, this study developed a multifunctional injectable hydrogel. The system is designed to prevent periodontal tissue destruction and restore a stable and healthy periodontal microenvironment, thereby offering a novel therapeutic strategy for periodontitis. 

## Methods

### Preparation of multifunctional injectable hydrogels

**Fig. 1:**
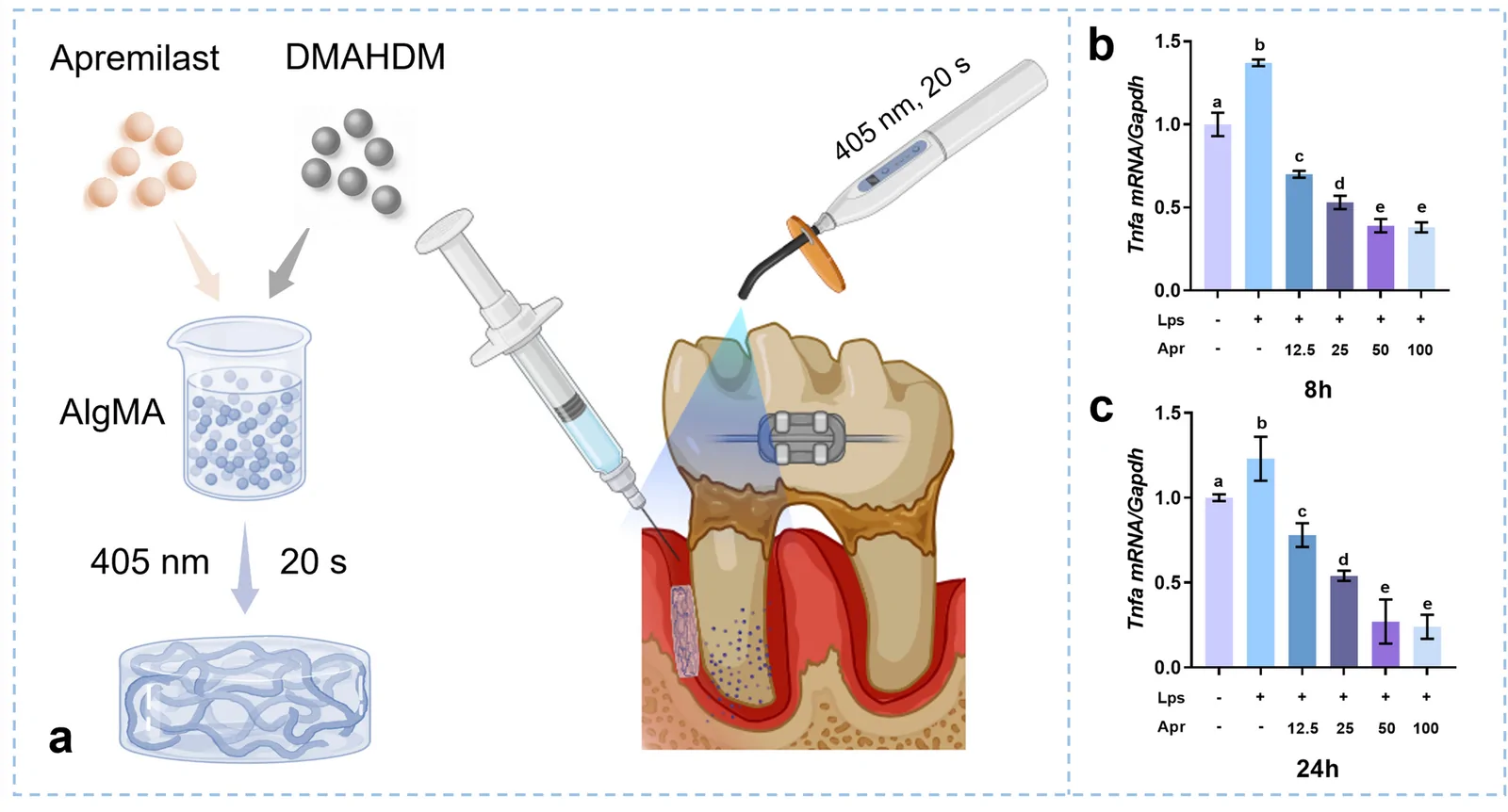
Synthesis of the multifunctional injectable hydrogel. **a** Schematic illustration of the hydrogel synthesis and its clinical application. **b** Effects of different concentrations of Apr on *Tnfa* mRNA expression in the in vitro inflammatory model after 8 h of treatment. **c** Effects of different concentrations of Apr on *Tnfa* mRNA expression in the in vitro inflammatory model after 24 h of treatment. Data are presented as mean ± SD (*n* = 3). Different lowercase letters above the bars indicate significant differences between groups (*P* < 0.05)

AlgMA (alginate methacryloyl) was purchased from EngineeringForLife (EFL, EFL-AlgMA-50k/300k) and dissolved according to the manufacturer’s instructions to prepare precursor solutions containing the photoinitiator LAP. DMAHDM and Apremilast (Apr) were subsequently added to the precursor solutions at different concentrations and thoroughly mixed. The resulting mixtures were placed at a distance of 15 mm from the light source and irradiated with 405 nm light (25 mW/cm², LED) for 20 s to induce photo-crosslinking, thereby forming the hydrogels (Fig. 1a).

DMAHDM was synthesized using a modified Menschutkin reaction, as described in our previous study [11]. Specifically, 2-(dimethylamino)ethyl methacrylate (DMAEMA) was used as the tertiary amine, while 1-bromohexadecane (BHD) served as the alkyl halide. In a 20 mL reaction vial, 10 mmol of DMAEMA (Tokyo Chemical Industry, Japan), 10 mmol of BHD (Monomer-Polymer and Dajac Labs, USA) and 3 g of ethanol were mixed. The vial was sealed and stirred at 70 °C for 24 h. After completion of the reaction, the ethanol was evaporated to obtain DMAHDM as a white solid.

Apremilast (Apr, IA4550) was purchased from Solarbio (Beijing, China). To determine an effective concentration for anti-inflammatory intervention, a range of Apr doses was evaluated by measuring the mRNA expression of the pro-inflammatory cytokine TNF-α using quantitative real-time PCR (qPCR) (Fig. 1b, c). The results showed that 50 µM Apr significantly downregulated *Tnf-α* expression, with no significant difference compared to 100 µM dose. Therefore, 50 µM was selected as the working concentration for subsequent hydrogel formulation.

Based on these findings, the experimental hydrogel groups were defined as follows: blank hydrogel; hydrogel containing 50 µM Apr; and hydrogels containing 50 µM Apr combined with DMAHDM at concentrations of 2.5, 5, 10, 20 and 40 µg/mL (Table 1).

**Table 1:** Experimental groups and hydrogel compositions for each group

Group	Compositions
Control	Hydrogel
Apr	Hydrogel + 50 µM Apremilast
Apr@2.5 µg/mL DMAHDM	Hydrogel + 50 µM Apremilast + 2.5 µg/mL DMAHDM
Apr@5 µg/mL DMAHDM	Hydrogel + 50 µM Apremilast + 5 µg/mL DMAHDM
Apr@10 µg/mL DMAHDM	Hydrogel + 50 µM Apremilast + 10 µg/mL DMAHDM
Apr@20 µg/mL DMAHDM	Hydrogel + 50 µM Apremilast + 20 µg/mL DMAHDM
Apr@40 µg/mL DMAHDM	Hydrogel + 50 µM Apremilast + 40 µg/mL DMAHDM

### Characterization of multifunctional injectable hydrogels

The prepared hydrogels were first extruded through a syringe to evaluate their injectability by observing flow behavior under applied force and shape retention. The cured hydrogel samples were then stored at -20 °C for 4 h, followed by -80 °C for 2 h, and subsequently lyophilized in a vacuum freeze-dryer for 18 h. The initial dry weight of the lyophilized samples was recorded for subsequent swelling and degradation assays.

Swelling behavior was evaluated by immersing the hydrogels in PBS (pH 7.4) and incubating at 37 °C, with weight changes recorded at 15, 30, 60, 120, 180, 240 and 300 min. The swelling ratio was calculated according to the following equation:

$$Swelling\;ratio\left(\%\right)=\frac{\left(W_{t}-W_{0}\right)}{W_{0}}\times{100}$$

where W_0_ is the initial dry weight of the hydrogel and W_t_ is the weight of the hydrogel at each time point.

For degradation analysis, the hydrogels were immersed in PBS (pH 7.4) containing type II collagenase (1.0 U/mL, Thermo Fisher Scientific, 17101015) and incubated at 37 °C. Samples collected at 1, 2, 3 and 4 weeks were dried and weighed to determine weight loss. The degradation rate was calculated using the following formula:

$$Degradation\;rate \left(\%\right)=\frac{\left(W_{i}-W_{d}\right)}{W_{i}}\times{100}$$

where W_i_ is the initial dry weight of the hydrogel and W_d_ is the remaining dry weight at each time point.

For mechanical testing, lyophilized samples (10 × 10 × 8 mm³) were subjected to compression using a universal testing machine (CMT6103, MTS Systems, China) at a crosshead speed of 1.0 mm/min. Stress-strain curves were recorded to determine the compressive modulus and evaluate the mechanical stability and tissue-supporting capability of the hydrogels.

Lyophilized samples (10 × 10 × 3 mm³) were gold-sputtered and observed under scanning electron microscopy (SEM, ZEISS GeminiSEM 300) to examine their three-dimensional network structure. During imaging, the accelerating voltage was set at 3 kV with a working distance of approximately 7.1 mm. Images were acquired at different magnifications (300× and 1000×) using a secondary electron (SE2) detector. The mean porosity was further quantified using ImageJ software (Version 1.53t, NIH, USA) based on the SEM micrographs.

### Evaluation of biocompatibility of multifunctional injectable hydrogels

In accordance with ISO 10993-5/12 standards, all hydrogel groups were sterilized and immersed in DMEM culture medium at 37 °C under appropriate humidity for 24 h to prepare hydrogel extracts. The L929 cell line, as a standard model for in vitro cytotoxicity evaluation, is widely used for biocompatibility assessment. As a fibroblast-like cell line, it can partially reflect the biological response of periodontal tissue cells to biomaterials, and was therefore selected for the biocompatibility evaluation in this study.

Healthy L929 fibroblasts (ATCC, CCL-1) were continuously passaged until reaching the logarithmic growth phase, then digested with trypsin, collected by centrifugation and resuspended in 2 mL of DMEM (hydrogel extracts) for accurate cell counting. Cells were subsequently seeded into 96-well plates at a density of 4,000 cells/well in 100 µL medium, with five replicate wells per group; wells containing only culture medium served as blank controls. The plates were incubated under standard conditions (37 °C, 5% CO₂) for 24, 48 and 72 h.

After incubation, 10 µL of CCK-8 reagent was added to each well under light-protected conditions, followed by incubation for an additional 2–4 h in the dark. The absorbance (optical density, OD) at 450 nm was then measured using a microplate reader and the mean OD value for each group was calculated. Relative cell viability was determined based on the OD values to evaluate the in vitro biocompatibility of the hydrogels.

### Assessment of the antibacterial properties of multifunctional injectable hydrogels

Periodontal pathogens *Porphyromonas gingivalis* (ATCC 33277, USA) and *Fusobacterium nucleatum* (ATCC 25586, USA) were obtained from the American Type Culture Collection (ATCC) and cultured under anaerobic conditions with repeated subculture until stable growth was achieved. The bacterial suspensions were adjusted to 1 × 10⁷ CFU/mL and co-incubated with the prepared hydrogel samples (10 × 10 × 3 mm³) for 48 h in a sealed anaerobic culture system (85% N₂, 5% H₂, 10% CO₂, 37 °C). After co-culture, the hydrogels were transferred to 15 mL centrifuge tubes containing 5 mL of PBS and the surface-associated biofilms were detached using an ultrasonic bath (40 kHz) for 3 min. The obtained suspensions were serially diluted, plated onto agar plates, and incubated under the same anaerobic conditions (85% N₂, 5% H₂, 10% CO₂, 37 °C) for 24 h. Colony-forming units (CFUs) were counted to quantitatively analyze the inhibitory effects of different hydrogels on *Porphyromonas gingivalis* and *Fusobacterium nucleatum*, thereby evaluating the in vitro antibacterial activity of each hydrogel group.

### Investigation of the anti-inflammatory and immunomodulatory activities of multifunctional injectable hydrogels

Multifunctional injectable hydrogels with confirmed biocompatibility and antibacterial activity were selected for immunomodulatory evaluation. The hydrogels were sterilized and immersed in DMEM at 37 °C for 24 h to obtain hydrogel extracts. RAW 264.7 macrophages (ATCC, TIB-71) were seeded in 6-well plates at a density of 2 × 10⁵ cells/well and stimulated with lipopolysaccharide (LPS, 1 µg/mL) for 12 h to establish an in vitro inflammatory model. The hydrogel extracts from each group were then co-cultured with the LPS-stimulated macrophages.

After incubation, the gene and protein expression levels of the pro-inflammatory cytokines TNF-α and IL-6 were analyzed by quantitative real-time PCR (qPCR) (CFX96, Bio-Rad) and enzyme-linked immunosorbent assay (ELISA, SpectraMax i3x, Molecular Devices) using TNF-α and IL-6 Mouse ELISA kits (EM0183 and EM0121, FineTest), respectively, to evaluate the anti-inflammatory effects of the hydrogels in vitro. The primer sequences used in this study were listed in Table 2.

**Table 2:** Oligonucleotide primers applied in real-time polymerase chain reaction

Primers	Nucleotide Sequence (5′-3′)
Gapdh	
Forward	5′- GGTTGTCTCCTGCGACTTCA -3′
Reverse	5′- GGTGGTCCAGGGTTTCTTACTC -3′
Tnfa	
Forward	5′- CCTGTAGCCCACGTCGTAGC -3′
Reverse	5′- TTGACCTCAGCGCTGAGTTG -3′
Il6	
Forward	5′- TGCTGGTGACAACCACGGCC -3′
Reverse	5′- GTACTCCAGAAGACCAGAGG -3′

To further investigate the effects of the hydrogels on macrophage phenotypes, RAW 264.7 cells were stained with antibodies against CD86 (GL1, BioLegend) and CD206 (C068C2, BioLegend), and macrophage polarization was analyzed by flow cytometry (BD Biosciences) to evaluate M1/M2 phenotype distribution.

### Exploring the angiogenesis induced by multifunctional injectable hydrogels

Human umbilical vein endothelial cells (HUVECs, ATCC, PCS-100-010) were seeded onto Matrigel-coated (Corning, 354234) 96-well plates at an initial density of 1 × 10⁴ cells/well. Different DMEM (hydrogel extract) were then added and co-cultured for 4 h. Tube formation was observed under a fluorescence microscope and images were analyzed using ImageJ software (Version 1.53t, NIH, USA). The images were converted to 8-bit grayscale and thresholded to remove background noise. The number of meshes and total tube length were quantified using the Angiogenesis Analyzer plugin. At least three random fields were analyzed for each group and the results were presented as Mean ± Standard.

After the tube formation assay, HUVECs were collected by digestion with 0.25% trypsin and total RNA was extracted and reverse-transcribed into cDNA. The mRNA expression level of VEGFA was analyzed by quantitative real-time PCR (qPCR). The culture supernatants were collected and centrifuged to remove cell debris and VEGFA protein levels were determined using an ELISA kit (EH0327, FineTest) according to the manufacturer’s instructions. The primer sequences used in this study were listed in Table 3.

**Table 3:** Oligonucleotide primers applied in real-time polymerase chain reaction

Primers	Nucleotide Sequence (5′-3′)
Gapdh	
Forward	5′- GGAGCGAGATCCCTCCAAAAT -3′
Reverse	5′- GGCTGTTGTCATACTTCTCATGG -3′
VEGFA	
Forward	5′- AGGGCAGAATCATCACGAAGT -3′
Reverse	5′- AGGGTCTCGATTGGATGGCA -3′

### Statistical analysis

All experimental data were analyzed using SPSS version 26.0 and presented as mean ± SD. Statistical significance was determined using one-way ANOVA followed by Tukey’s HSD post hoc test. Each experiment was performed with at least three independent biological replicates and each biological replicate included three technical replicates. *P* < 0.05 was considered statistically significant.

## Results

It was illustrated in Fig. 1a that the multifunctional injectable hydrogel was synthesized and applied as described. As shown in Fig. 1b and c, in the LPS-induced inflammatory model, the control group treated with LPS alone exhibited high *Tnfa* mRNA expression levels at both 8 and 24 h. With intervention using different concentrations of Apr (12.5, 25, 50 and 100 µM), *Tnfa* mRNA expression decreased in a dose-dependent manner. Notably, the 50 µM group reached a plateau, showing no significant difference compared with the 100 µM group, indicating that this concentration achieved effective anti-inflammatory effects. Further increasing the concentration did not provide additional benefit; therefore, 50 µM was selected as the Apr concentration for incorporation into the hydrogel.

**Fig. 2:**
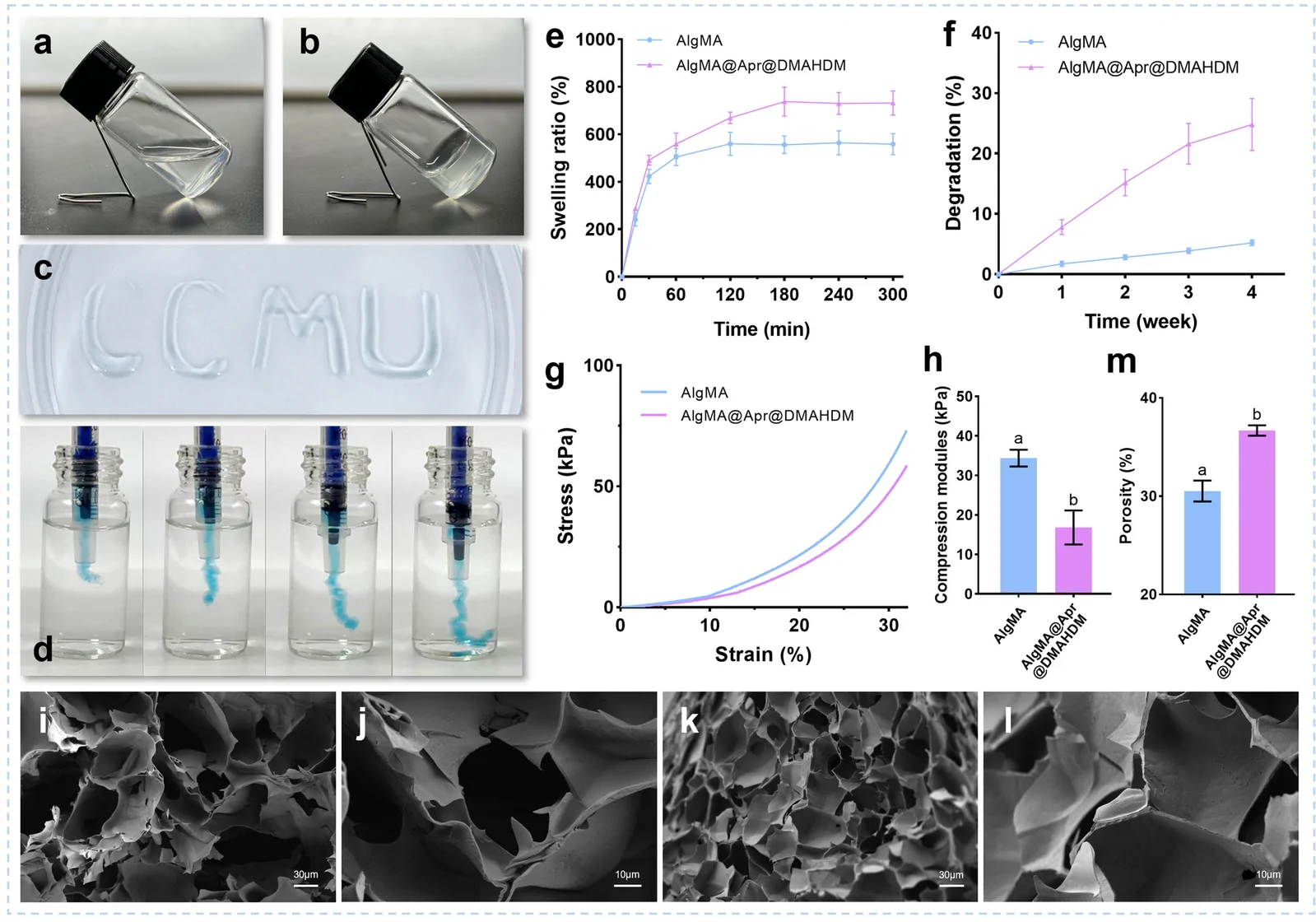
Characterization of the multifunctional injectable hydrogel. **a** The hydrogel precursor solution appears as a transparent, homogeneous liquid before light exposure. **b** Upon exposure to 405 nm light for 20 s, the solution undergoes rapid gelation, exhibiting good self-supporting properties and stable shape retention. **c** Optical image of the hydrogel extruded from a syringe to write “CCMU”. **d** Hydrogel injectability and in situ gelation in an aqueous environment; the extruded gel filament retained its structural integrity and shape without significant diffusion. **e** Swelling behavior. The composite hydrogel exhibited a significantly enhanced swelling capacity. **f** In vitro degradation. Cumulative mass loss over a 4-week period; the composite hydrogel showed accelerated degradation, consistent with its higher swelling ratio. **g** Compressive stress-strain curves. Representative profiles illustrating the characteristic nonlinear mechanical behavior of the swollen polymer networks. **h** Compressive modulus. The composite hydrogel exhibited a significantly lower modulus, indicating improved mechanical compliance, favorable for soft tissue regeneration. **i**-**l** Scanning electron microscopy (SEM) images (300× and 1000× magnifications) revealed that the composite hydrogel possessed an interconnected 3D porous network characterized by a looser and more open architecture. **m** Quantitative analysis of surface porosity indicated that the composite hydrogel had significantly higher porosity. Data are presented as mean ± SD (*n* = 3). Different lowercase letters above the bars indicate significant differences between groups (*P* < 0.05)

As shown in Fig. 2a, the hydrogel precursor solution was initially liquid, transparent and homogeneous. Upon exposure to 405 nm light for 20 s, the solution rapidly gelled (Fig. 2b), exhibiting excellent self-supporting properties and maintaining its shape for a certain period. In Fig. 2c and d, the injectability of the hydrogel was demonstrated by extruding it to write the letters “CCMU.” After being injected into water, the hydrogel maintained its shape without noticeable diffusion, indicating that the synthesized hydrogel possessed excellent injectable properties.

The swelling and degradation behaviors of the hydrogels were systematically evaluated under simulated physiological conditions. As shown in Fig. 2e, the AlgMA@Apr@DMAHDM group reached an equilibrium swelling ratio of approximately 740% after 180 min, significantly higher than that of the AlgMA group. Consistent with its swelling behavior, the composite hydrogel exhibited accelerated degradation (Fig. 2f). Up to 4 weeks, the cumulative mass loss of the AlgMA@Apr@DMAHDM group was approximately 24.8%, whereas the AlgMA group remained largely stable, exhibiting less than 6% degradation.

As shown in Fig. 2g, both hydrogel groups displayed typical nonlinear stress-strain curves. The composite hydrogel exhibited a lower slope at the initial stage and a notably lower maximum stress compared with the AlgMA group. The compressive modulus, calculated from the initial linear portion of the curves, was presented in Fig. 2h. The composite hydrogel showed a modulus of approximately 17 kPa, considerably lower than 34 kPa measured for the AlgMA group. This was mainly attributed to electrostatic repulsion between the quaternary ammonium groups in DMAHDM, which expanded the polymer network and improved the hydrogel’s hydration and injectability.

The internal microstructure and mechanical properties of the hydrogels were evaluated by SEM and quantitative analysis (Fig. 2i and m). Both AlgMA and AlgMA@Apr@DMAHDM hydrogels exhibited interconnected 3D porous networks; however, the incorporation of Apr and DMAHDM resulted in a looser and more open structure compared with the AlgMA group (Fig. 2k and l). ImageJ analysis confirmed that the composite hydrogel had significantly higher porosity than the AlgMA group (*P* < 0.05) (Fig. 2m), consistent with its increased swelling behavior (Fig. 2e). This suggested that the introduced components partially reduced the crosslinking density of AlgMA, leading to a more porous network.

**Fig. 3:**
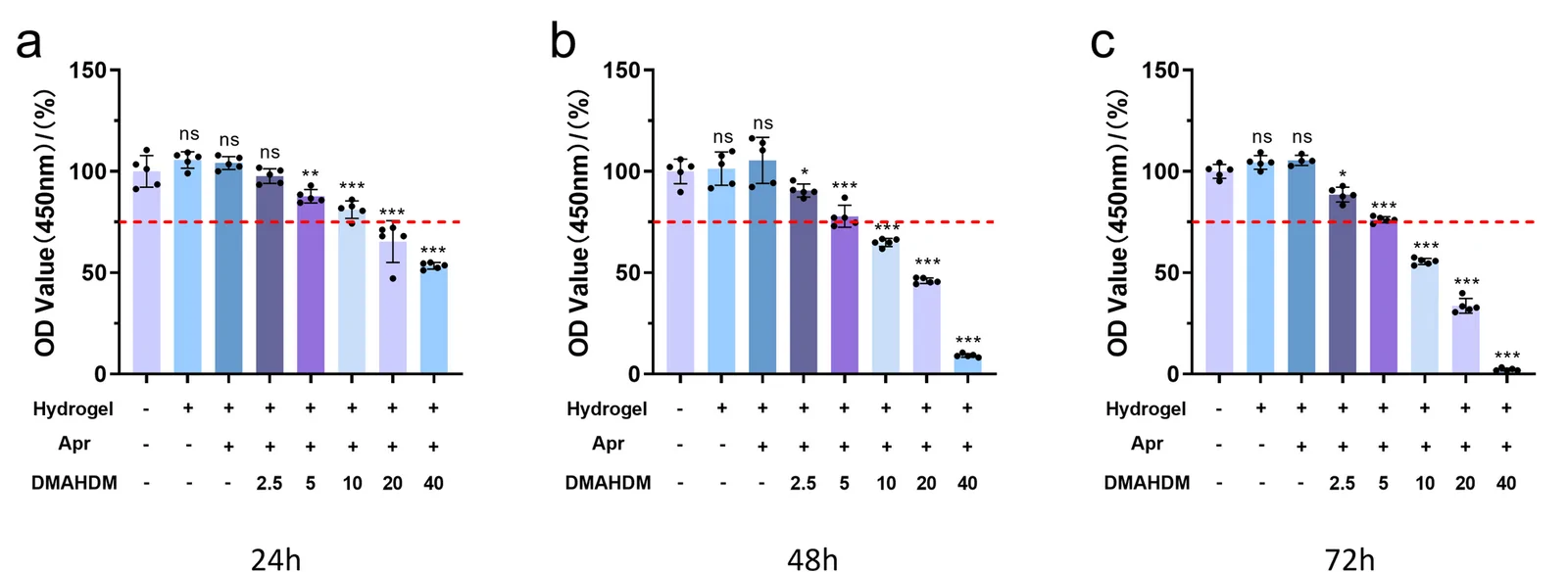
Biocompatibility of the multifunctional injectable hydrogel. **a**-**c** Relative proliferation of L929 cells treated with hydrogel extracts for 24, 48 and 72 h. The hydrogel containing 50 µM Apr@5 µg/mL DMAHDM showed no obvious cytotoxicity, indicating good biocompatibility. Data are presented as mean ± SD (*n* = 5). Compared with the Blank (leftmost) group, “ns” denotes no significant difference (*P*>0.05), while “*” indicate statistical significance (*: *P*<0.05; **: *P*<0.01; ***: *P*<0.001)

Biocompatibility was evaluated by assessing the relative proliferation of L929 cells after co-culture with hydrogel extracts from different groups for 24, 48 and 72 h (Fig. 3). In the Apr-treated group, cell viability was 105.7 ± 4.03%, 101.4 ± 8.19% and 104.5 ± 3.39% at the three time points, all exceeding the 75% threshold defined by ISO standards, indicating that Apr had no detrimental effect on the biocompatibility of the composite hydrogel. In contrast, the presence of DMAHDM exhibited a concentration-dependent impact on cell viability. Specifically, proliferation in the Apr@10 µg/mL DMAHDM group fell below 75% at 48 and 72 h, whereas the Apr@5 µg/mL DMAHDM group maintained viability above the safety threshold at all time points.

**Fig. 4:**
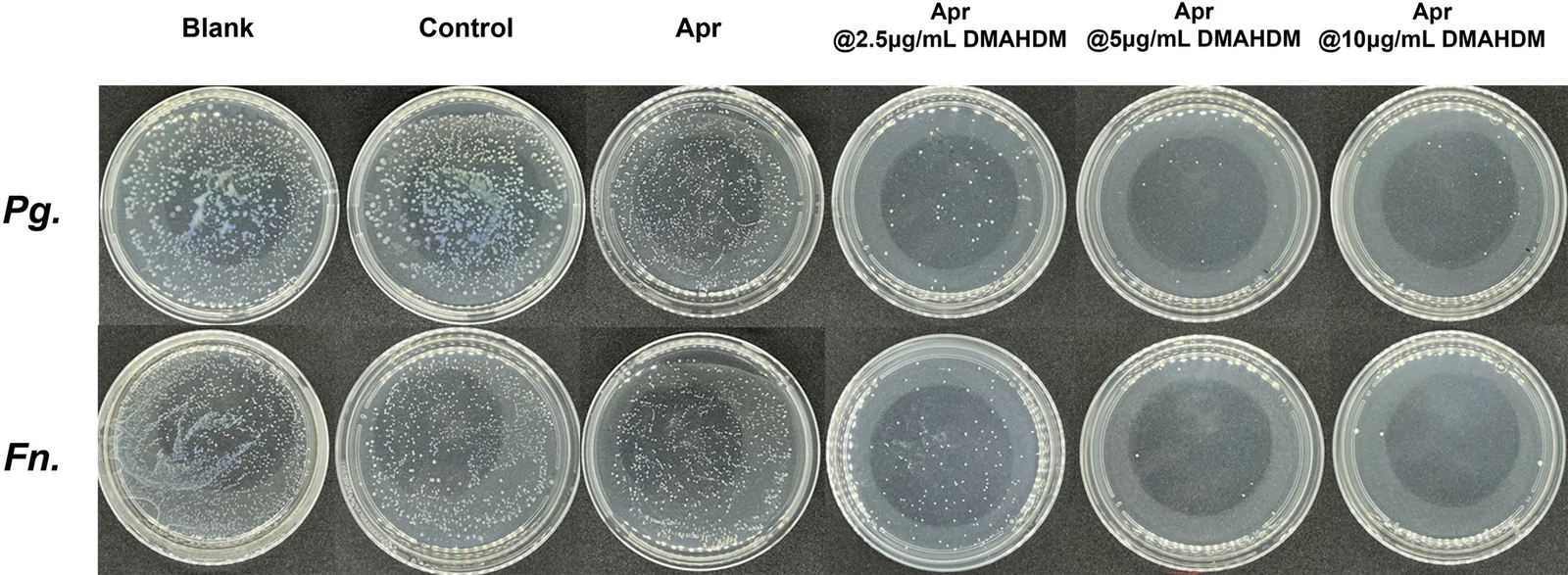
Antibacterial activity of the multifunctional injectable hydrogel. Representative colony formation assays illustrating the inhibitory effects of different hydrogel treatments on *Porphyromonas gingivalis* (Pg) and *Fusobacterium nucleatum* (Fn). Extensive colony growth was observed in the Blank and Control groups. While Apr monotherapy demonstrated a moderate inhibitory effect, the composite hydrogels exhibited progressively enhanced antibacterial effects with increasing DMAHDM concentrations (2.5, 5 and 10 µg/mL)

In the evaluation of antibacterial activity (Fig. 4), the composite hydrogel exhibited pronounced inhibitory effects against *Porphyromonas gingivalis* and *Fusobacterium nucleatum*. Compared with treatment with Apr alone, incorporation of DMAHDM significantly enhanced the antibacterial efficacy of the hydrogels. Notably, the hydrogel containing 5 µg/mL DMAHDM demonstrated stronger antibacterial activity than the 2.5 µg/mL group, indicating a dose-dependent effect of DMAHDM on antibacterial performance. Based on these findings, the in vitro anti-inflammatory activity of each hydrogel group was performed to determine a formulation with a favorable balance of biocompatibility, antibacterial efficacy and anti-inflammatory effects.

**Fig. 5:**
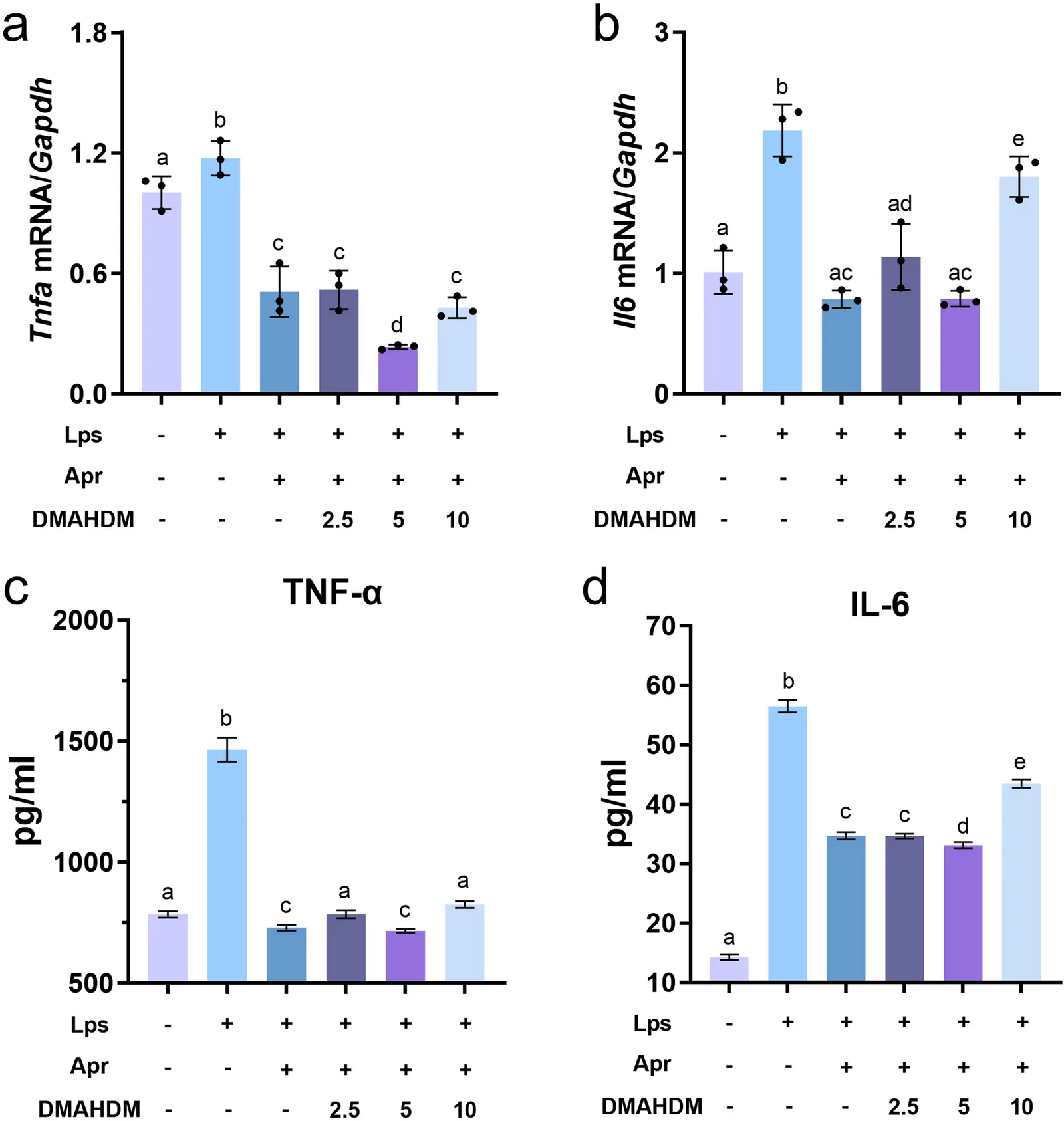
Anti-inflammatory effects of the multifunctional injectable hydrogel. **a** qPCR analysis of *Tnfa* mRNA expression. **b** qPCR analysis of *Il6* mRNA expression. **c** ELISA measurement of TNF-α protein levels. **d** ELISA measurement of IL-6 protein levels. Both qPCR and ELISA results demonstrated that the Apr@5 µg/mL DMAHDM hydrogel exerted the most pronounced anti-inflammatory effect. Data are presented as mean ± SD (*n* = 3). Different lowercase letters above the bars indicate significant differences between groups (*P* < 0.05)

As shown in Fig. 5, LPS stimulation markedly upregulated *Il6* and *Tnfa* mRNA expression and promoted the secretion of TNF-α and IL-6 proteins. In comparison, treatment with Apr alone significantly suppressed the expression of these inflammatory mediators at both the transcriptional and protein levels. Incorporation of DMAHDM into the composite hydrogel further enhanced this inhibitory effect. Among the tested DMAHDM concentrations, the Apr@5 µg/mL DMAHDM group showed the most pronounced suppression of *Il6* and *Tnfa* mRNA expression as well as TNF-α and IL-6 secretion (*P* < 0.05), indicating superior anti-inflammatory efficacy under the current experimental conditions. Accordingly, 5 µg/mL DMAHDM was selected for subsequent experiments due to its favorable balance of biocompatibility, antibacterial activity and anti-inflammatory properties.

**Fig. 6:**
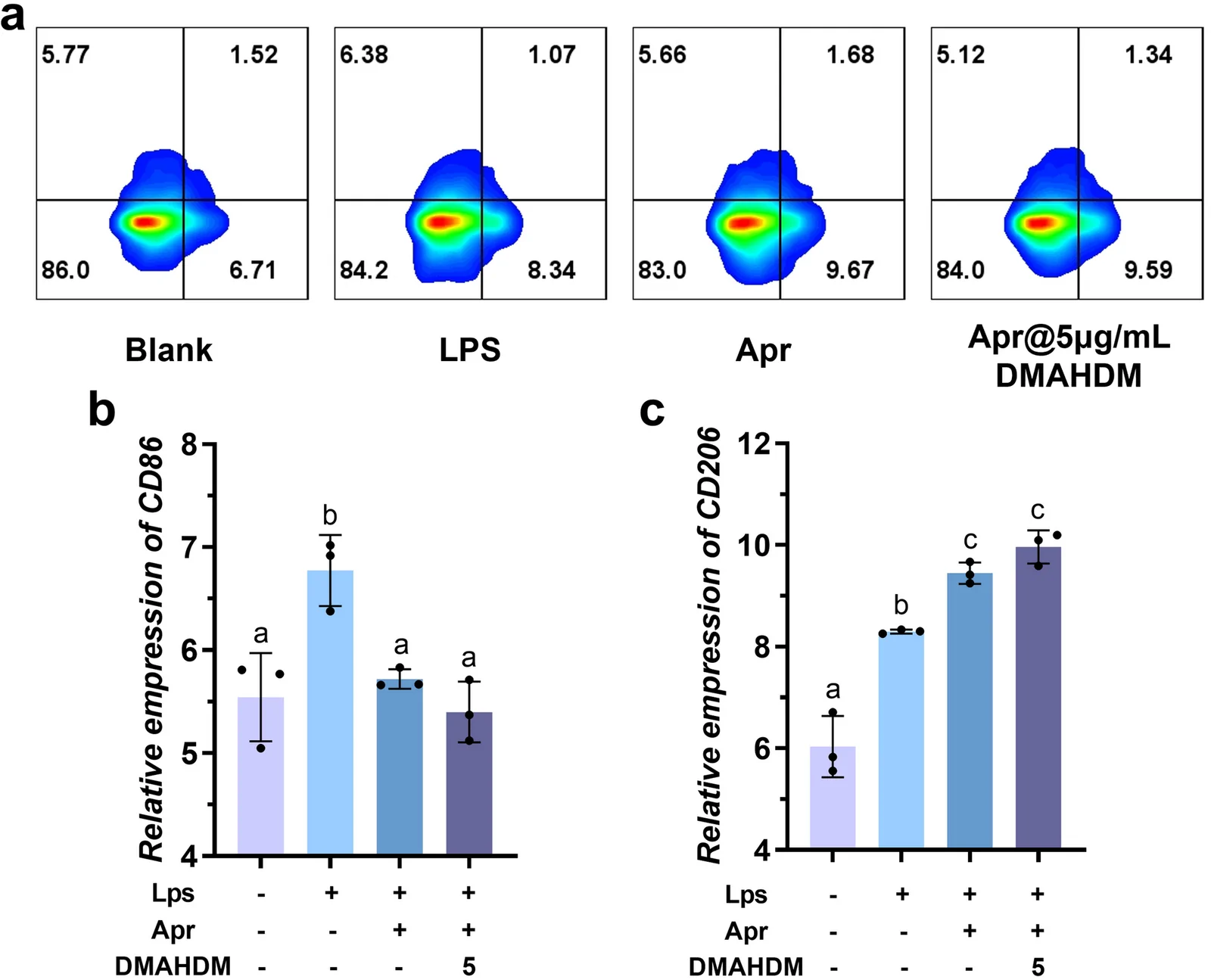
Regulation of macrophage phenotype polarization by the multifunctional injectable hydrogel. **a** Flow cytometric analysis of macrophage surface markers CD86 and CD206 (upper-left: CD86; lower-right: CD206). **b** Quantification of relative CD86 expression. **c** Quantification of relative CD206 expression. LPS stimulation significantly upregulated CD86 expression, promoting M1 pro-inflammatory polarization. In contrast, treatment with the Apr@5 µg/mL DMAHDM hydrogel markedly decreased CD86 expression and increased CD206 expression, thereby promoting macrophage polarization toward the M2 anti-inflammatory phenotype. Data are presented as mean ± SD (*n* = 3). Different lowercase letters above the bars indicate significant differences between groups (*P* < 0.05)

As illustrated in Fig. 6, the regulatory effects of the Apr@5 µg/mL DMAHDM hydrogel on macrophage phenotypes were systematically evaluated. LPS stimulation induced a strong CD86 signal, indicative of M1 macrophages, whereas treatment with the hydrogel markedly reduced CD86 expression. Concurrently, the CD206 signal, representing M2 macrophages, was significantly enhanced, indicating that the hydrogel not only suppressed the pro-inflammatory M1 population but also promoted polarization toward the anti-inflammatory, tissue-repairing M2 phenotype, highlighting its potential immunomodulatory function.

**Fig. 7:**
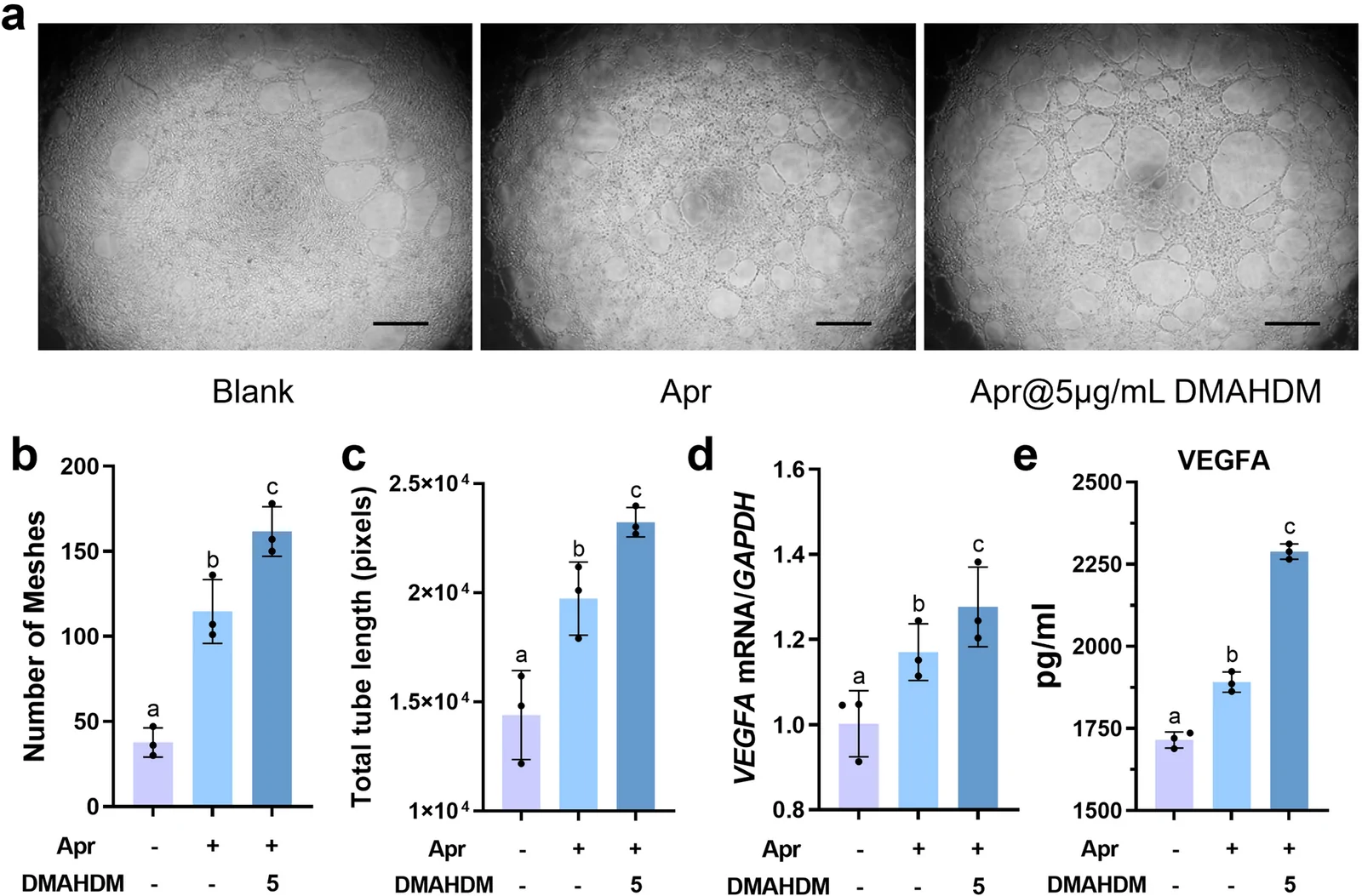
Angiogenic effects of the multifunctional injectable hydrogel. **a** Representative images of HUVEC tube formation in the Matrigel assay. **b**,** c** Quantitative analysis of tube formation, including mesh number and total tube length. **d** Relative *VEGFA* mRNA expression assessed by RT-qPCR. (e) VEGFA protein levels in culture supernatants measured by ELISA. Data are presented as mean ± SD (*n* = 3). Different lowercase letters above the bars indicate significant differences between groups (*P* < 0.05)

Matrigel tube formation assays illustrated that, compared with the Blank control group, Apr-treatment promoted endothelial tube formation, whereas the Apr@5 µg/mL DMAHDM treatment group exhibited a more pronounced effect, characterized by denser mesh structures and more continuous tubular networks (Fig. 7a). Quantitative analysis further demonstrated that the Apr@5 µg/mL DMAHDM group exhibited a significant increase in both number of meshes and total tube length (Fig. 7b-c), which was statistically significant compared with both the Blank control and Apr-monotherapy groups (*P* < 0.05). qPCR and ELISA analyses demonstrated that Apr upregulated *VEGFA* mRNA expression and protein levels, and this effect was further enhanced by DMAHDM conjugation (Fig. 7d-e, *P* < 0.05). Additionally, Collectively, these results indicated that the composite hydrogel demonstrated preliminary pro-angiogenic activity in vitro, suggesting its potential role in supporting vascular-related processes during tissue repair.

## Discussion

The central pathological feature of periodontitis is a persistent inflammatory response driven by pathogenic microbial colonization and dysregulation of host immune homeostasis, forming a self-amplifying vicious cycle between bacterial colonization and immune imbalance [13]. Conventional therapies often fail to achieve both effective microbial eradication and precise control of inflammation, thereby limiting therapeutic efficacy. Although mechanical debridement or antimicrobial monotherapy can reduce plaque burden, these approaches are usually insufficient to resolve the hyperactive immune-inflammatory response, leading to progressive destruction of periodontal supporting tissues [14].

Building on the pathological mechanisms described above, this study developed a multifunctional local delivery platform by incorporating the phosphodiesterase-4 (PDE4) inhibitor Apr and the quaternary ammonium antibacterial agent DMAHDM into an alginate methacryloyl (AlgMA) injectable hydrogel to achieve synergistic antibacterial, anti-inflammatory, and immunomodulatory effects [15]. AlgMA, a methacrylated derivative of natural alginate [16], possesses excellent biocompatibility and photo-crosslinking properties. Under 405 nm irradiation, the precursor solution gelled rapidly (within approximately 20 s) in the presence of the photoinitiator LAP. This property enables injection into irregular periodontal pockets followed by in situ solidification, thereby allowing precise modulation of the local periodontal microenvironment.

The photo-crosslinking system used in the present study has been widely applied in dental light-curable materials and has a well-established clinical safety profile. Short-duration irradiation (20 s) enables rapid gelation while minimizing local temperature rise and reducing the risk of cytotoxicity associated with light exposure. However, in the context of deep periodontal pockets, effective light penetration may still be limited. To improve clinical feasibility, slender light-curing tips or fiber-optic guides can be employed to deliver light into the periodontal pocket, ensuring complete hydrogel polymerization in situ [17]. It should be noted that the present study is currently limited to in vitro experiments, and the distribution of light irradiation, temperature changes during photo-crosslinking and potential photobiological effects have not yet been systematically evaluated in the complex oral environment. Future studies will focus on optimizing the photoinitiator system to enhance curing efficiency in deep periodontal defects and validating thermal safety, biocompatibility, and clinical feasibility in animal models.

Physicochemical and mechanical analyses suggested that the incorporation of DMAHDM altered the AlgMA network via electrostatic repulsion between quaternary ammonium groups, resulting in a more loose and porous three-dimensional structure [11]. This structural change likely explained the pronounced increase in swelling capacity and the excellent injectability observed in the complex periodontal environment. Although this microstructural expansion reduced the compressive modulus, it may indicate enhanced mechanical compliance of the hydrogel, thereby facilitating conformity to surrounding soft tissues. In addition, increased accessibility of water and enzymes to the polymer backbone facilitated a swelling-driven degradation process, enabling responsive drug release and supporting long-term management of periodontal inflammation.

Biocompatibility is a fundamental prerequisite for the clinical translation of biomedical materials [18]. According to ISO 10993-12 guidelines, the cytocompatibility of the hydrogel system was evaluated using L929 fibroblast proliferation assays. The results showed that the hydrogel loaded with 50 µM Apr did not induce significant cytotoxicity over 24–72 h, demonstrating good compatibility with the AlgMA matrix. Although increasing the concentration of DMAHDM enhanced the antibacterial efficacy, cell viability decreased markedly with prolonged culture when the concentration reached ≥ 10 µg/mL, suggesting that high levels of DMAHDM may adversely affect cytocompatibility. In contrast, 5 µg/mL DMAHDM maintained favorable cytocompatibility while providing significant antibacterial and anti-inflammatory effects in vitro. Therefore, this concentration was identified as a favorable balance and selected for subsequent experiments to further explore its multifunctional properties.

The present study demonstrated that the synthesized hydrogel exhibited significant inhibitory effects against the major periodontal pathogens *Porphyromonas gingivalis* and *Fusobacterium nucleatum* [19]. Apr alone showed limited antibacterial activity, whereas the incorporation of DMAHDM markedly enhanced antibacterial efficacy, achieving near-complete inhibition at 5 µg/mL. The antibacterial activity is primarily attributed to the cationic quaternary ammonium groups, which disrupt the electrostatic balance and compromise the integrity of bacterial cell membranes. In addition, the long alkyl chains of DMAHDM can insert into the lipid bilayer, further enhancing membrane disruption and bactericidal efficiency, thereby enabling a sustained contact-killing effect [20]. Previous studies have demonstrated that DMAHDM exhibits stable and long-lasting antibacterial properties in dental materials such as resin composites and adhesives [10]. The present findings further support the feasibility of incorporating DMAHDM into injectable hydrogel-based delivery systems.

It should be noted that, although the in vitro CFU assays demonstrated significant inhibitory effects of the hydrogel against *Porphyromonas gingivalis* and *Fusobacterium nucleatum*, the antibacterial model used in this study remains relatively simple, being based solely on single-species cultures. In clinical conditions, periodontitis typically involves structurally complex multispecies biofilms [21], whose highly organized architecture and enhanced resilience may influence the in vivo efficacy of antimicrobial agents. Therefore, future studies will employ periodontal-relevant multispecies biofilm models or in vitro polymicrobial culture systems to more accurately simulate the periodontal microenvironment and evaluate the long-term antibacterial potential of the hydrogel under complex biofilm conditions.

Excessive release of pro-inflammatory cytokines and dysregulated macrophage polarization are recognized as key mechanisms driving the persistent destruction of periodontal tissues [22]. In the present study, qPCR and ELISA analyses showed that treatment with the composite hydrogel significantly reduced both the gene and protein expression levels of TNF-α and IL-6, indicating a potential anti-inflammatory effect. Mechanistically, this effect is likely attributable to the inhibition of PDE4 by Apr, which elevates intracellular cAMP levels and modulates downstream inflammation-related signaling pathways, potentially including PKA and NF-κB [23]. Consequently, transcription of pro-inflammatory genes is suppressed, while the expression of anti-inflammatory mediators is enhanced, contributing to the improvement of the local inflammatory microenvironment [24].

Furthermore, the cAMP/PKA signaling pathway plays a key role in macrophage polarization [25]. Elevated intracellular cAMP levels can promote the polarization of macrophages from M1 phenotype to M2 phenotype, thereby reducing the release of inflammatory cytokines and facilitating tissue regeneration. In the present study, flow cytometry analysis showed that the hydrogel treatment decreased the expression of the M1 marker CD86 while markedly upregulating the M2 marker CD206, indicating effective promotion of macrophage polarization toward an anti-inflammatory, reparative phenotype. M2 macrophages not only suppress inflammatory responses but also secrete various growth factors, thereby promoting periodontal ligament cell proliferation and alveolar bone regeneration.

It should be noted that the inference of anti-inflammatory effects in this study is primarily based on the previously reported pharmacological mechanism of Apr as a selective PDE4 inhibitor [23–25]. However, intracellular cAMP levels and the activity of downstream signaling molecules were not directly assessed, representing a limitation of the present study. Future studies will integrate molecular biology and signaling pathway analyses to systematically elucidate the hydrogel’s effects on inflammation suppression, immune modulation and remodeling of the periodontal microenvironment, thereby further clarifying its multi-target regulatory potential and providing stronger experimental evidence for its application in late-stage periodontal tissue regeneration.

Angiogenesis plays a critical role in tissue repair by supplying oxygen and nutrients, thereby creating favorable conditions for the proliferation and differentiation of periodontal ligament cells, fibroblasts and osteoblasts [26]. In vitro angiogenesis assays showed that hydrogel treatment increased both the density and continuity of HUVEC tubular networks and upregulated VEGFA mRNA and protein expression, suggesting that the hydrogel may promote endothelial tube formation and provide preliminary evidence for its potential vascular-supporting role in the periodontal microenvironment. Moreover, the observed promotion of endothelial cell proliferation further indicated the favorable biocompatibility of the hydrogel. It should be noted, however, that these results were obtained in vitro and may not fully reflect the hydrogel’s effects on periodontal tissue regeneration in vivo. Future studies will employ animal models to systematically assess its impact on local angiogenesis, periodontal ligament and alveolar bone reconstruction, as well as to further elucidate the underlying mechanisms, thereby validating its therapeutic potential for periodontal tissue regeneration.

In summary, the injectable AlgMA hydrogel loaded with 50 µM Apr and 5 µg/mL DMAHDM demonstrated good in vitro biocompatibility while exhibiting preliminary antibacterial, anti-inflammatory and immunomodulatory potential. This combinatorial approach partially overcomes the limitations of conventional local periodontal treatments, which often struggle to simultaneously control bacterial infection and inflammatory responses and provides a novel strategy for improving the local microenvironment in periodontitis. It should be emphasized, however, that in vitro experiments cannot fully replicate the complex oral microenvironment Therefore, further animal studies are required to systematically evaluate the hydrogel’s in vivo performance, thereby providing more robust experimental evidence to support its potential clinical translation.

## Conclusions

We successfully developed an injectable multifunctional hydrogel loaded with Apr and DMAHDM, exhibiting good biocompatibility and effective inhibition of periodontal pathogens *Porphyromonas gingivalis* and *Fusobacterium nucleatum in vitro*. It also reduced the expression of pro-inflammatory cytokines TNF-α and IL-6 and modulated macrophage polarization, indicating potential anti-inflammatory and immunoregulatory effects and providing a theoretical basis for subsequent in vivo and mechanistic studies. Further investigations are necessary to evaluate its performance and therapeutic potential in vivo.

## Data Availability

The datasets used and/or analysed during the current study are available from the corresponding author on reasonable request.
